# Prevalence of Multidrug-Resistant *Escherichia coli* Isolated from Drinking Water Sources

**DOI:** 10.1155/2018/7204013

**Published:** 2018-08-19

**Authors:** Stephen T. Odonkor, Kennedy K. Addo

**Affiliations:** ^1^Greenhill College, Ghana Institute of Management and Public Administration, Accra, Ghana; ^2^Department of Bacteriology, Noguchi Memorial Institute for Medical Research, University of Ghana, Accra, Ghana

## Abstract

The control of infectious diseases is badly endangered by the rise in the number of microorganisms that are resistant to antimicrobial agents. This is because infections caused by resistant microorganisms often fail to respond to conventional treatment, resulting in prolonged illness and greater risk of death. Antimicrobial-resistant bacteria are also present in various water sources. This study therefore sought to document the microbiological quality and antibiograms of bacterial isolates (*E. coli* strains) from six different water sources in order to determine their safety for human consumption and to provide updated antibiotic data for pragmatic treatment of patients. Bacteria isolation and identification was done using API and conventional methods. Antibiotic susceptibility testing was conducted using the Kirby–Bauer method. Results obtained indicated that all the water sources tested were of poor quality. Bacteria isolated included *E. coli*, *Enterobacter* spp., *Klebsiella* spp., *Salmonella typhi*, *Streptococcus* spp., *Proteus vulgaris*, *Vibrio cholera*, *Shigella* spp., *Pseudomonas aeruginosa*, and *Enterococcus faecalis*. The prevalence of multidrug-resistant *E. coli* was 49.48%. *E. coli* isolates showed high resistance patterns to the tested antibiotics. They were most resistant to penicillin (32.99%), cefuroxime (28.87%), erythromycin (23.71%), and tetracycline (21.45%). In contrast, they were susceptible to nitrofurantoin (93.8%), cefotaxime and amikacin (91.75%), gentamicin (90.7%), nalidixic acid (89.65%), ciprofloxacin (74.2%), chloramphenicol (69.07%), pipemidic acid (65.97%), and cefuroxime (52.58%). Sixty-three percent (63%) of the multidrug-resistant *E. coli* strains recorded a multiple antibiotic resistance (MAR) index value >0.2. The susceptible antibiotics, especially the nitrofurantoin, are hence recommended in the practical treatment of waterborne bacterial diseases.

## 1. Introduction

Antibiotics are arguably the most successful form of chemotherapy developed in the 20th century and save innumerable human lives every day [1]. The emergence of antibiotic-resistant bacteria limits the clinical use of antibiotics and, as resistant bacteria become more prevalent, there is increasing concern that existing antibiotics will become ineffective against these pathogens and more expensive [2].

Antibiotic-resistant genes conferring resistance to a wide variety of antibiotics have been identified in a large range of water environments including drinking water in both developed and developing countries [3, 4]. The main risk for public health is that resistance genes are transferred from environmental bacteria to human pathogens. The potential of drinking water to transport microbial pathogens to a greater number of people, causing subsequent illness, is well documented in countries at all levels of economic development [5, 6]. Furthermore, the availability of safe drinking water is an indispensible feature for preventing epidemic disease and improving the quality of life [7, 8]. According to the World Health Organization, 80% of all diseases are attributed to unsafe water [9]. Developing countries in particular, are plagued with water-related diseases such as diarrhoea which account for 10% of the disease burden in such countries [10].

*Escherichia coli* is a member of feacal coliforms that contaminate drinking water from human and animal feacal waste. *E. coli* has been the foremost indicator of faecal contamination in water quality monitoring for many decades. During rainfalls, these coliforms may be washed into creeks, rivers, streams, lakes, or ground water. Untreated drinking water coming from these sources contains coliforms including *E. coli*.

*E. coli* has also been shown to be a significant reservoir of genes coding for antimicrobial drug resistance and therefore is a useful indicator for resistance in bacterial communities [11, 12]. Although there are several studies assessing multidrug resistance (MDR) in *E. coli* populations of animal origin, not much work has been done on the ecology of MDR [13, 14]. The spread of MDR into environments where antibiotics are not used is a possibility that has not yet been well researched, although it has been postulated that water could disseminate antimicrobial resistance [15]. The objectives of this study are to determine the antibiotic sensitivity pattern and the multiple antibiotic resistance index of *E. coli* strains isolated from six drinking water sources during bacteriological monitoring over a year.

## 2. Materials and Methods

### 2.1. Sample Collection Sites

After several preliminary visits to various communities in the districts, 57 sampling sites comprising six different water sources that include dams, boreholes, stream sources, rivers, canals, and hand-dug wells in 27 communities were selected. Samples were taken from locations that were representative of the water sources and/or distribution networks from which water is delivered to the inhabitants and/or points of use based primarily on factors such as population and extent of usage or level of patronage of water from these sources. Most of the communities are dominated by farmers. Each community selected had at least a borehole or a stream as the principal sources of water for the inhabitants.

### 2.2. Site Observation Details

Prior to water sampling, important observations were made around the sampling sites. These observations included the sanitary conditions as well as possible sources of contamination, which could influence water quality from the sources sampled.

Field records for the following environmental factors were also recorded: water clarity/turbidity (visual clarity in the water, i.e., leaves, debris, and algae), weather conditions (temperature, wind, and rainfall), presence of animals (birds/ducks), and other comments (e.g., system problems, i.e., disinfection/filtration equipment, and faecal accidents).

### 2.3. Sample Size and Sampling Frequency

A total of one hundred twenty-two water samples were collected for assessment between June 2011 and May 2012. The sample collection period spanned over the two seasons in Ghana, that is, the dry and rainy seasons. All water sampling and preservation procedures were performed according to Standard Methods for the examination of water and wastewater [16, 17] and WHO guidelines for drinking water quality [14, 15]. Sampling for bacteriological analysis was done aseptically with care, ensuring no external contamination of samples. All samples were transported to the laboratory within 2 hours.

### 2.4. Bacteria Isolation and Identification

All Gram-positive organisms were identified by conventional methods such as Gram stain, positive catalase, tube coagulase, deoxyribonucleases (DNAse) test, and so on, while an API 20E kit was used to identify the Gram-negative organism. *E. coli* strain 25922 was used as the positive control for the *E. coli* isolates.

### 2.5. Antibacterial Susceptibility Testing of *E. coli*

Each of the isolates (*E. coli*) was subjected to antibiotic susceptibility testing using the Kirby–Bauer method that has been standardized and evaluated by the methods of the National Committee for Clinical Laboratory Standards [18]. Isolates grown overnight on Nutrient Agar were suspended in sterile normal saline (0.9% w/v NaCl) using a sterile wire loop until the turbidity was equivalent to 0.5 Mcfarland standards. Sterile nontoxic cotton swabs dipped into the standardized inocula were used to streak the entire surface of Mueller–Hinton agar plates. The *E. coli* isolates were then tested against fourteen antibiotics as follows: ampicillin (10 *μ*g), pipemidic acid (20 ug), chloramphenicol (30 *μ*g), ciprofloxacin (5 *μ*g), co-trimoxazole (25 *μ*g), erythromycin (15 *μ*g), nitrofurantoin (300 *μ*g), penicillin (10 IU), cefuroxime (30 *μ*g), cefotaxime (30 *μ*g), nalidixic acid (30 *μ*g), amikacin (30 *μ*g), tetracycline (30 *μ*g), and gentamicin (10 *μ*g). Antibiotic disks were aseptically placed using sterile forceps, and all plates were incubated (Gallenkamp England, model IH-150) at 37°C for 24 hrs [19]. The results were interpreted using NCCLS [18].

## 3. Results

Results from Table 1 shows that a total of five hundred twenty bacterial isolates (520) were obtained during the period of study. A significant number of the isolates (305) representing 58.65% of the total were obtained during the dry season, as against (205) representing 41.35% in the rainy season.

The most commonly occurring organism in the water samples was *Klebsiella* spp. (104), representing 20% of the total number of isolates obtained. The highest number of *Klebsiella* spp. (18) was isolated from stream water sources during the dry season and the lowest (1) from rivers during the rainy season. The next most occurring organism was *E. coli* (97), representing 18.7% of the total bacterial isolates. This was followed by *Pseudomonas aeruginosa* (15.61%), *Enterobacter* spp. (15.4%), *Proteus vulgaris* (13.1%), and *Enterococcus faecali* (9.2%). The least isolated organism was *Vibrio cholerae* (1.2%) and *Shigella* spp. (1.2%). *Vibrio cholerae* was isolated in four water sources, namely, stream, borehole, hand-dug wells, and dam water sources, while *Shigella* spp. was isolated in 3: stream, borehole, and dam water sources.

A total of one hundred twenty-two water samples were collected for the bacteriological analysis. Results from Table 2 show that ninety-seven *E. coli* strains were isolated during the period of the study. Fifty-eight strains representing 59.79% were isolated during the dry season as against thirty-nine representing 40.21% in the rainy season. The highest number of strains isolated from a single water source was from dams (28) representing 29%. This was followed by stream water sources (26) representing 27%, hand-dug wells (24) representing 25%, and borehole water sources (11) representing 11%. River water sources produced the least number of isolated strains (2) representing 2% and then canal water sources (6) representing 6%. The highest isolates during the rainy season were obtained from dams (12) followed by stream water sources (10) and hand-dug wells (10). The highest number of isolates during the dry season were obtained from dams (16) followed by stream water sources (17). The least number of isolates during the rainy season were obtained from canals (3) followed by borehole water sources (4). No *E. coli* strain was isolated from river water sources. The least number of isolates during the dry season were obtained from rivers (2) followed by canal water sources (3).

Results from Table 3 reveal the antibiotic susceptibility profile of the *E. coli* strains. All the strains were tested against 14 different antibiotics, using Kirby–Bauer disc diffusion, standardized and evaluated by the methods of National Committee for the Clinical Laboratory Standards [18]. Table 3 shows that *E. coli* strains were most resistant to penicillin (32) representing 32.99%, followed by cefuroxime (28) representing 28%, erythromycin (23) representing 23.71%, tetracycline (21) representing 21.45%, chloramphenicol (18) representing 18.65%, pipemidic acid (13) representing 13.40%, and ampicillin (11) representing 11.32%. Seven out of the fourteen antibiotics had ten or less number of isolates showing resistance. Four isolates representing 4.12% were resistant to each of the following antibiotic: cefotaxime, nalidixic acid, and nitrofurantoin. This was followed by gentamicin (5) representing 5.15%, amikacin (7) representing 7.2%, ciprofloxacin (8) representing 8.5%, and finally co-trimoxazole (8) representing 8.5%. Table 3 shows that *E. coli* strains were most susceptible/sensitive to nitrofurantoin (91) representing 93.8%, and this was followed by cefotaxime and amikacin (89) representing 91.75%, gentamicin (88) representing 90.7%, nalidixic acid (87) representing 89.65%, ciprofloxacin (72) representing 74.2%, chloramphenicol (67) representing 69.07%, pipemidic acid (64) representing 65.97%, and cefuroxime (CXM) (51) representing 52.58%. Four out of the fourteen antibiotics had fifty or less number of isolates showing resistance. They were penicillin (14), tetracycline (29), ampicillin (45), and erythromycin (50).

Analysis of multiple drug resistance of *E. coli* isolates from the water sources reveals that forty-eight isolates representing large percentage of (49.48%) of *E. coli* isolates exhibited resistance against two or more antibiotics, thus classified as multidrug resistance. This creates a huge public health concern.

## 4. Discussion

The presence of *E. coli* in the various water sources may spell health hazards such as diarrhoeal diseases which account for a substantial degree of morbidity and mortality in adults and children [20–24]. Control of diarrhoea may require the administration of antibiotics. Nonetheless, several strains of *E. coli* are known to be resistant to a wide array of antibiotics [25–28]. Multiple antibiotic resistances refer to resistance to either two or more classes of antibiotics. The multiple antibiotic resistances of *E. coli* established in this study agree with other findings [29–34]. Strains of *E. coli* and *Salmonella* spp. accounted for several outbreaks in the United States and worldwide, partly due to resistance to chloramphenicol, ampicillin, and trimethoprim [35, 36].

The frequency of penicillin resistance in the current study was high among the isolates as compared with chloramphenicol and ampicillin resistance observed in the isolates obtained from the various water sources. This may be due to the blanket use of inexpensive antibiotics in the Ghanaian community or may be due to production of beta-lactamase enzymes. *E. coli* resistance against ampicillin was observed by Çelebi et. al. [37], Olowe et al. [35], and Yurdakoek et al. [34]. The emerging co-trimoxazole and ciprofloxacin resistance from downstream sites are of serious concern, as these are the preferred drugs for many Gram-negative bacteria [33]. The most common mechanism of resistance to co-trimoxazole is the acquisition of plasmid-mediated, variant diaminopyrimidine folate reductase enzymes [38]. Low resistance to amikacin and gentamycin might be due to the less use of these antibiotics in clinical practice and/or veterinary medicine. The rising trend of resistance in all the isolates (total and faecal coliforms) from upstream to downstream affirms the fact that disposed antibiotics may have been washed down the water sources and accumulated downstream especially during the rainy season accounting for the high resistance.

The differences in resistance profiles in this environmental study clearly reflect the differences in the selection procedure pressure in the investigated sites/areas. The higher level of resistance to antibiotics among coliforms of midstream and downstream sites of Ghanaian communities is worrisome since most inhabitants take bath, wash clothes, and even disposed human sewage into water sources at midstream and downstream sites while some occupants and nonoccupants use these water sources for drinking and/or domestic purposes. In Mangalore, it is reported that untreated or partially treated domestic sewage is released into open estuaries which accounts for the high level of antibiotic resistance [39].

Multidrug resistance is defined as resistance to all the tested antibiotics in at least two of the following three classes: lactams, aminoglycosides, and quinolones [39]. The multidrug resistance (MDR) characters of the isolates were identified by observing the resistance pattern of the isolates to the antibiotics. The MAR index of an isolate is defined as *a/b*, where *a* represents the number of antibiotics to which the isolate was resistant and *b* represents the number of antibiotics to which the isolate was subjected [40]. The MAR index analysis reveals that thirty of the multidrug-resistant *E. coli* strains had a very high MAR index value (>0.2). The high MAR index recorded in this study relates the fact that the water sources may have been highly contaminated with antibiotics due to the high usage of these chemicals in the surrounding areas of the various water sources. This is in accordance with the Tambekar et al.'s [39] report which states that bacteria originating from an environment where several antibiotics are used usually produce MAR index greater than 0.2. MAR indexing below 0.2 determined in this study was actually below the illogical value of risk contamination [41]. However, samples that yielded MAR indexing above 0.2 indicated high risk of contamination. The difference in MAR indexing in the different water sources indicated the impact of urbanization on antibiotic resistance levels.

Microbiological quality of the various water sources analyzed was low as diverse bacterial strains were isolated with different frequencies. To a greater extent, differences in antibiotic resistance frequencies were detected in *E. coli* strains from different water sources such that some *E. coli* strains were highly resistant to cefotaxime, nalidixic acid, nitrofurantoin, gentamicin, amikacin, ciprofloxacin, and co-trimoxazole. The differences in antibiotic strains of the various water sources could reflect the specific use of antibiotics around the specified source. The high prevalence of penicillin and chloramphenicol resistance recorded poses a serious public health concern since these antibiotics stand less chance of curing infected patients who use the surveyed water sources as drinking water or for domestic purposes. Indeed, the increasing prevalence of resistance in the isolates, especially, of the human origin, may have an important therapeutic implication that calls for caution in the indiscriminate use of antibiotics on humans. However, nearly all the 97 strains of *E. coli* were susceptible to some antibiotics, namely, nitrofurantoin (93.8%), followed by cefotaxime and amikacin (91.75%), gentamicin (90.7%), nalidixic acid (89.65%), ciprofloxacin (74.2%), chloramphenicol (69.07%), pipemidic acid (65.97%), and lastly by cefuroxime (52.58%).

High and low MAR index values were recorded in the study, which indicates the level of risk of contamination of the sampled water sources, which call for more restrictive policies on the disposal of human/animal sewages and bathing/washing in or close to water bodies. Lastly, periodic monitoring of antibiotic sensitivity of the water sources is of importance to detect any changing patterns that may arise in future in order to keep pace with such changing patterns for better curative measures or policies formulation and implementation.

## Figures and Tables

**Table 1:** Distribution of different species of bacteria isolates from water samples.

Bacteria	Dams		Boreholes		Streams		Hand-dug wells		Rivers		Canals		Total (%)
	Rainy	Dry	Rainy	Dry	Rainy	Dry	Rainy	Dry	Rainy	Dry	Rainy	Dry	
*E. coli*	12	16	4	7	10	16	10	14	0	2	3	3	97 (18.7)
*Enterobacter* spp.	11	15	2	6	8	13	8	13	0	1	1	2	80 (15.4)
*Klebsiella* spp.	12	16	4	6	15	18	10	14	1	2	3	3	104 (20.0)
*Salmonella typhi*	0	2	2	2	1	2	1	2	0	0	0	1	13 (2.5)
*Streptococcus* spp.	2	7	0	0	1	1	2	3	0	0	1	0	17 (3.3)
*Proteus vulgaris*	10	12	2	5	10	14	5	9	0	0	0	1	68 (13.1)
*Vibrio cholerae*	1	0	0	1	2	1	1	0	0	0	0	0	6 (1.2)
*Shigella* spp.	1	0	0	0	2	3	0	0	0	0	0	0	6 (1.2)
*Pseudomonas aeruginosa*	10	12	2	6	12	14	8	11	1	2	1	2	81 (15.6)
*Enterococcus faecali*	5	3	2	3	9	8	5	8	1	1	1	2	48 (9.2)
Total	64 (12.3)	83 (16.0)	18 (3.5)	36 (6.9)	70 (13.5)	90 (17.3)	50 (9.6)	74 (14.2)	3 (0.6)	8 (1.5)	10 (0.10)	14 (0.7)	520 (100)

**Table 2:** Frequency of isolation of *E. coli* strains in the rainy and dry season.

Water sources	Number of samples analyzed		Number of strains of *E. coli* isolated		Total (%)
	Rainy	Dry	Rainy	Dry	
Dams	15	15	12	16	28 (29)
Boreholes	8	8	4	7	11 (11)
Streams	17	17	10	16	26 (27)
Hand-dug wells	15	15	10	14	24 (25)
Rivers	3	3	0	2	2 (2)
Canals	3	3	3	3	6 (6)
Total	61	61	39	58	97 (100)

**Table 3:** Antibiotic resistance patterns of *E. coli* isolates from the various water sources.

Antibiotic	Susceptibility			
	Disc concentration	Resistant number (%)	Intermediate number (%)	Sensitive number (%)
Amikacin (AMK)	30 *μ*g	7 (7.22)	1 (1.03)	89 (91.75)
Ampicillin (AMP)	10 *μ*g	11 (11.32)	41 (42.27)	45 (46.39)
Cefotaxime (CTX)	30 *μ*g	4 (4.12)	4 (4.12)	89 (91.75)
Cefuroxime (CXM)	30 *μ*g	28 (28.87)	18 (18.65)	51 (52.58)
Chloramphenicol (CHL)	30 *μ*g	18 (18.56)	12 (12.37)	67 (69.07)
Ciprofloxacin (CIP)	5 *μ*g	8 (8.25)	17 (17.53)	72 (74.22)
Co-trimoxazole (COT)	25 *μ*g	10 (10.31)	6 (6.19)	81 (83.50)
Erythromycin (ERY)	15 *μ*g	23 (23.71)	24 (24.74)	50 (51.55)
Gentamicin (GEN)	10 *μ*g	5 (5.15)	4 (4.12)	88 (90.72)
Nalidixic acid (NAL)	10 *μ*g	4 (4.12)	6 (6.19)	87 (89.69)
Nitrofurantoin (NIT)	300 *μ*g	4 (4.12)	2 (2.060)	91 (93.81)
Penicillin (PEN)	10 units	32 (32.99)	51 (52.58)	14 (14.43)
Pipemidic acid (PA)	20 *μ*g	13 (13.40)	20 (20.62)	64 (65.98)
Tetracycline (TET)	30 *μ*g	21 (21.45)	47 (48.45)	29 (29.90)

## Data Availability

The data used to support the findings of this study are available from the corresponding author upon request.
